# Herpes Zoster in Patients Treated with JAK Inhibitors for Immune-Mediated Inflammatory Diseases: Incidence, Associated Factors and Vaccination Uptake in a Real-World Cohort

**DOI:** 10.3390/jcm15103733

**Published:** 2026-05-13

**Authors:** António Parchão, Carolina Monteiro, Leonardo Araújo-Andrade, Cláudia Camila Dias, Cândida Abreu

**Affiliations:** 1 Faculty of Medicine, University of Porto, Alameda Prof. Hernâni Monteiro, 4200-319 Porto, Portugal; carolina.v.monteiro@ulssjoao.min-saude.pt (C.M.); laandrade@med.upt.pt (L.A.-A.); candida.abreu@ulssjoao.min-saude.pt (C.A.); 2 Infectious Diseases Department, Unidade Local de Saúde São João, Alameda Prof. Hernâni Monteiro, 4200-319 Porto, Portugal; 3 NeuroGen Research Group, Center for Health Technology and Services Research (CINTESIS), Rua Dr. Plácido da Costa, 4200-450 Porto, Portugal; 4 RISE-Health, Unit of Anatomy, Department of Biomedicine, Faculty of Medicine, University of Porto, Alameda Prof. Hernâni Monteiro, 4200-319 Porto, Portugal; 5 Knowledge Management Unit, Department of Community Medicine, Information and Health Decision Sciences, Faculty of Medicine, University of Porto, Alameda Prof. Hernâni Monteiro, 4200-319 Porto, Portugal; camila@med.up.pt; 6 RISE-Health, Department of Community Medicine, Information and Health Decision Sciences, Faculty of Medicine of the University of Porto, Alameda Prof. Hernâni Monteiro, 4200-319 Porto, Portugal; 7 RISE-Health, Department of Medicine, Faculty of Medicine, University of Porto, Alameda Prof. Hernâni Monteiro, 4200-319 Porto, Portugal

**Keywords:** Janus kinase inhibitors, immune-mediated inflammatory diseases, herpes zoster, recombinant zoster vaccine

## Abstract

**Background/Objectives:** This study aimed to determine the incidence of herpes zoster (HZ) and risk factors associated with its occurrence in patients receiving Janus kinase inhibitors (JAKis) for immune-mediated inflammatory diseases (IMIDs), while evaluating preventive strategies and zoster vaccine uptake. **Methods:** We conducted a retrospective single-center cohort study including patients with rheumatoid arthritis, psoriatic arthritis, ankylosing spondylitis, inflammatory bowel disease, atopic dermatitis and alopecia areata treated with upadacitinib, baricitinib or tofacitinib. The primary outcome was incident HZ during JAKi exposure. Incidence rates (IRs) were calculated per 100 person-years (PY) and Cox regression identified factors associated with HZ. **Results:** A total of 292 patients contributed 565.5 PY of JAKi exposure. During follow-up, 23 patients (7.9%) developed HZ, corresponding to an overall IR of 4.07/100 PY (95% CI 2.40–5.73). Incidence rates were numerically lower with upadacitinib and varied across disease groups; differences were not statistically significant. Diabetes mellitus (HR 3.05, 95% CI 1.28–7.29) and chronic kidney disease (HR 3.24, 95% CI 1.17–8.95) were independently associated with HZ. Herpes simplex infection requiring systemic antiviral therapy was more frequent among patients who developed HZ. Recombinant zoster vaccine (RZV) uptake was low (9.9%), but higher among patients evaluated in a dedicated infectious risk consultation. No HZ events were observed among RZV-vaccinated patients. Although six HZ events (26.1%) were severe, all cases resolved completely. **Conclusions:** HZ remains a relevant complication of JAKi therapy across IMIDs. Diabetes mellitus and chronic kidney disease may help identify higher-risk patients, while structured infectious risk assessment could improve vaccine uptake.

## 1. Introduction

In recent years, Janus kinase inhibitors (JAKis) have become an increasingly established therapeutic class for the treatment of several immune-mediated inflammatory diseases (IMIDs), including rheumatoid arthritis, axial and peripheral spondyloarthritis, inflammatory bowel disease and selected dermatological conditions [1]. These agents act by inhibiting the Janus kinase-signal transducer and activator of transcription (JAK-STAT) pathway, a key intracellular cascade for multiple pro-inflammatory cytokines, which is known to be overactivated in these conditions [2]. By targeting shared pathogenic mechanisms, JAKis provide effective disease control. Their oral administration and rapid onset of action have further supported their widespread use in clinical practice [3].

Despite its clinical benefits, JAK inhibition has been associated with an increased susceptibility to infections. Among these, herpes zoster (HZ) has been consistently highlighted across clinical trials and real-world studies [4,5,6].

Herpes zoster results from the reactivation of latent varicella-zoster virus (VZV), with the risk increasing during periods of impaired cell-mediated immunity [7,8]. Typical presentation involves a painful unilateral vesicular rash confined to a single dermatome [8,9]. In addition to acute morbidity, up to a third of patients may develop postherpetic neuralgia, which can significantly impair quality of life [7,8,9]. More severe presentations may occur, including ophthalmic, disseminated, neurological or visceral involvement, particularly in immunocompromised patients [8,10].

In the general population, the incidence of HZ is estimated at three to five cases per 1000 person-years (PY) [10,11]. Patients with IMIDs have a baseline risk up to threefold higher [12,13] and the risk is further increased in those treated with JAKis [6]. In a recent systematic review with network meta-analysis encompassing various IMIDs, incidence rates (IRs) ranged from approximately 1.5 to 4 cases per 100 PY, varying by underlying disease and specific agent [14]. JAK inhibitors seem to exhibit dose and possibly time-dependent patterns of HZ risk [14,15,16,17], which has also been hypothesized to be inversely associated with JAK1 selectivity [5,12]. Simultaneously, patient-related factors, namely advanced age, female sex, Asian ethnicity, prior HZ history and concomitant high-dose glucocorticoid exposure, contribute to overall risk [17,18,19,20,21].

However, much of the available evidence on HZ risk derives from cohorts of patients with rheumatoid arthritis, with comparatively limited real-world data across other IMIDs. Comparative data across underlying diagnosis and individual JAKi exposure in real-world settings are also scarce and require further study [14,15,17,22].

Prevention of VZV reactivation is largely achievable through vaccination. The introduction of a recombinant zoster vaccine (RZV) has significantly improved prevention strategies in immunosuppressed populations, offering superior efficacy and safety compared with the previous live attenuated vaccine [13,23]. Despite the robust evidence supporting its use, vaccination coverage among JAKi-treated patients remains suboptimal in some settings [15,21,24], potentially reflecting financial barriers in healthcare systems and lack of reimbursement policies [13,25,26].

The present study aimed to evaluate the incidence and clinical predictors of HZ in a real-world cohort of patients treated with upadacitinib, baricitinib and tofacitinib across a range of IMIDs. A secondary aim was to assess zoster vaccination uptake and prevention strategies in clinical practice. In doing so, this study seeks to address the need for broader real-world evidence, particularly including underrepresented patient groups and offering insight into preventive approaches.

## 2. Materials and Methods

### 2.1. Study Design and Setting

This was a retrospective, single-center cohort study conducted at a tertiary academic hospital in Portugal. Data were collected from outpatient pharmacy dispensing records and electronic health records (EHRs). The study period spanned from March 2014, corresponding to the first recorded exposure to JAKi at our institution, to 24 September 2025.

### 2.2. Study Population

All patients with a documented diagnosis of rheumatoid arthritis, Crohn’s disease, ulcerative colitis, ankylosing spondylitis, psoriatic arthritis, atopic dermatitis or alopecia areata who had been prescribed upadacitinib, baricitinib or tofacitinib on or before 31 December 2024 were eligible for inclusion. Patients were excluded if the prescribed JAKi was not initiated or if essential follow-up data for the primary outcome or exposure assessment were unavailable or incomplete.

Where patients had more than one eligible diagnosis, the primary diagnosis was defined as the condition identified by the treating specialist as the indication prompting JAKi initiation, consistent with the prescribed dosing regimen.

For subgroup analyses, patients were classified according to primary diagnosis into four broader groups: rheumatoid arthritis, spondyloarthritis (ankylosing spondylitis and psoriatic arthritis), inflammatory bowel disease (Crohn’s disease and ulcerative colitis) and dermatological diseases (atopic dermatitis and alopecia areata). This grouping was adopted to enhance interpretability and avoid sparse data in smaller subgroups.

### 2.3. Variables and Outcome Definition

Data retrieved from EHRs included sex, age at JAKi initiation, primary diagnosis and relevant comorbidities, namely diabetes mellitus and chronic kidney disease (CKD), defined as an estimated glomerular filtration rate < 60 mL/min/1.73 m^2^ persisting for at least 3 months during follow-up.

History of prior HZ and the occurrence of clinically significant herpes simplex virus (HSV) infection during follow-up were also recorded. HSV infection was defined as documented episodes requiring systemic antiviral therapy.

Additional variables included JAKi treatment characteristics, concomitant immunosuppressive or immunomodulatory therapies, attendance at a dedicated infectious risk consultation (IRC) and HZ vaccination status.

The primary outcome was incident HZ, defined as a clinically documented episode during JAKi exposure. Secondary outcomes included HZ complications, post-event management, uptake of preventive measures and recurrence.

Severe HZ was defined as any episode requiring hospital admission or presenting with disseminated disease, bacterial superinfection, or ophthalmic, neurologic or visceral involvement. Disseminated HZ was defined as involvement of more than two non-contiguous dermatomes. Postherpetic neuralgia was recorded separately and defined as neuropathic pain persisting for at least 3 months following the HZ episode.

For comorbidities, prior HZ and HZ-related complications, in the absence of documentation in EHRs, were assumed to indicate an absence of the condition. No imputation methods were used.

### 2.4. Exposure Assessment

Effective exposure time to JAKis was calculated from treatment initiation (index date) until HZ diagnosis, treatment discontinuation, death or study censoring, whichever occurred first, and was expressed in PY.

Some patients contributed more than one non-overlapping JAKi exposure period due to treatment interruption with subsequent reinitiation or switching between inhibitors. For IR calculations, person-time exposure was attributed to the JAKi administered during each exposure period and HZ events were assigned to the agent in use at the time of diagnosis.

### 2.5. Statistical Analysis

Categorical variables were presented as counts and percentages, and continuous variables were presented as the median with interquartile range (IQR). Demographic, clinical and treatment characteristics were compared according to HZ occurrence. Separate comparisons were made with respect to uptake of at least one dose of RZV.

Group comparisons were conducted using the Chi-square or Fisher’s exact test for categorical variables, as appropriate, and the Mann–Whitney U test was used for continuous variables.

Herpes zoster IRs were calculated for the overall cohort and stratified by JAKi and disease group, expressed as the number of zoster events per 100 PY of JAKi exposure, with corresponding 95% confidence intervals (CIs).

A multivariable Cox proportional hazards regression was performed to evaluate factors associated with HZ occurrence. Given the limited number of events, the final model was restricted to selected clinically relevant covariates informed by univariate analysis, ultimately including diabetes mellitus and CKD. Results were reported as hazard ratios (HR) with 95% CIs.

A two-tailed *p*-value < 0.05 was considered significant for all statistical tests. Statistical analyses were performed using IBM SPSS Statistics Version 30.

## 3. Results

### 3.1. Study Cohort Characteristics

A total of 301 patients met the eligibility criteria. Nine were excluded due to non-initiation of treatment or incomplete follow-up data, resulting in a final cohort of 292 patients. These patients contributed a cumulative 565.5 PY of JAKi exposure. The median exposure time per patient was 546.5 days (IQR 330.8–919.0).

The median age at JAKi initiation was 45 years (IQR 31–56) and 188 patients (64.4%) were female.

According to primary diagnosis, 110 patients (37.7%) had rheumatoid arthritis, 91 (31.2%) had inflammatory bowel disease, 54 (18.5%) had dermatological diseases, including atopic dermatitis (*n* = 38) and alopecia areata (*n* = 16), and 37 (12.7%) had spondyloarthritis, including ankylosing spondylitis (*n* = 20) and psoriatic arthritis (*n* = 17).

During the follow-up period, 23 patients (7.9%) developed HZ.

### 3.2. Incidence of HZ

The overall IR of HZ was 4.07 events per 100 PY (95% CI: 2.40–5.73).

Incidence rates were numerically lower among patients treated with upadacitinib (3.22/100 PY) compared to baricitinib and tofacitinib, although CIs overlapped (Table 1).

Incidence rates also varied across disease groups. The highest rate was observed in the spondyloarthritis group (7.51/100 PY), whereas the lowest rate was observed in patients with inflammatory bowel disease (2.51/100 PY). However, CIs were wide and overlapped substantially (Table 2).

### 3.3. Factors Associated with HZ

Patients’ demographic, clinical and treatment characteristics according to HZ occurrence are summarized in Table 3.

Patients with HZ were slightly older and more frequently female, but neither difference was statistically significant. Prior history of HZ also showed a non-significant trend toward higher prevalence among those who developed HZ during follow-up (13.0% vs. 4.1%, *p* = 0.088).

Patients who developed HZ had a significantly higher prevalence of diabetes mellitus compared with those who did not (34.8% vs. 11.2%, *p* = 0.005). Similarly, CKD was strongly associated with HZ occurrence (26.1% vs. 3.7%, *p* < 0.001), with 6 out of the 16 patients diagnosed with CKD developing HZ. Herpes simplex infection requiring systemic antiviral therapy was more frequently documented in patients with HZ occurrence (17.4% vs. 4.1%, *p* = 0.022).

The distribution of primary disease groups did not differ significantly between patients with and without HZ. The distribution of patients according to specific diagnosis is presented in Supplementary Table S1. None of the 19 patients with a concurrent additional IMID diagnosis developed HZ infection during follow-up.

No significant associations were established between HZ occurrence and treatment variables, including the initial JAKi, number of JAKis, duration of JAKi exposure, number of concomitant immunosuppressants and corticosteroid exposure. Concomitant immunosuppressive therapies across groups are detailed in Supplementary Table S2.

In a multivariable Cox regression analysis (Table 4), both CKD (HR 3.24, *p* = 0.023) and diabetes mellitus (HR 3.05, *p* = 0.012) were independently associated with incident HZ.

### 3.4. Vaccination Uptake and Preventive Assessment

Herpes zoster vaccination was prescribed to 51 patients (17.5%), predominantly the recombinant type. Of these, 32 (62.7%) were ultimately vaccinated: 29 received at least one RZV dose, 26 (89.7%) of whom completed the two-dose schedule, and 3 received a live attenuated vaccine. Only 19 prescriptions (37.2%) and 12 administrations (40.0%) occurred prior to JAKi initiation. Nearly half (49.0%) were prescribed vaccination during a dedicated IRC.

Patients’ characteristics according to RZV vaccination status are summarized in Table 5. Vaccination uptake was lower among female patients and higher among those with inflammatory bowel disease, while age did not differ significantly between groups.

Evaluation in an IRC in the context of JAKi initiation occurred in 89 patients (30.5%) and was significantly associated with higher RZV uptake (18.0% vs. 6.4%, *p* = 0.02), particularly when the consultation took place before treatment initiation (*p* < 0.001) (Table 5).

No HZ events were observed among patients who received at least one dose of RZV during 26.1 PY of exposure following vaccination (with a median of 305 days, IQR 141–407). Among the three patients vaccinated with the live attenuated vaccine, one developed HZ 106 days after vaccine administration.

### 3.5. Clinical Characteristics, Management and Recurrence of HZ

Among the 23 HZ cases included, the median age at diagnosis was 52 years (IQR 39–62). Median duration of continuous JAKi exposure prior to the event was 847 days (IQR 245–978), ranging from 60 to 3605 days.

The JAKi and respective doses at the time of diagnosis are included in Supplementary Table S3. No drug or dose-dependent pattern was identified.

Six cases (26.1%) were classified as severe, and one of them required hospital admission. The characteristics and complications of severe cases are explored in Table 6. Additionally, three patients (13.0%) developed postherpetic neuralgia.

Temporary interruption of JAKi therapy following VZV reactivation was reported in two patients, and permanent discontinuation was reported in two others.

All HZ cases resolved completely, with no documented long-term sequelae.

Following HZ, 10 patients (43.5%) were assessed in IRC. A total of eight patients (34.8%) were subsequently prescribed RZV, of whom seven were part of the IRC. All patients completed the two-dose schedule.

During a cumulative 27.0 PY of JAKi exposure after HZ, among twenty-one patients, one unvaccinated patient experienced two non-complicated recurrences of HZ (Table 6).

## 4. Discussion

In this real-world cohort of patients with IMIDs treated with JAKi, HZ occurred at an IR of 4.07 events per 100 PY, consistent with previous estimates [13,14,17,20,27,28,29,30]. This finding reinforces HZ as a relevant safety concern associated with this pharmacological class. Unlike many prior studies, our cohort included patients spanning multiple specialties and indications, thereby better reflecting contemporary prescription patterns and enabling the assessment of shared risk factors and preventive strategies across disease groups. Overall incidence appeared similar across the different drugs and disease groups, supporting the concept of a class effect [17], although some variability was observed.

Antiviral immune responses are largely dependent on type I (IFN-α/β) and type II (IFN-γ) interferons, which inhibit viral replication and promote innate and adaptive immune responses, such as natural killer and CD8+ T-cell activity via the JAK-STAT pathway [17,29,31,32]. Interestingly, varicella-zoster virus is known to evade host immunity by disrupting IFN-induced JAK-STAT signaling, thereby facilitating viral replication and persistence [17,31]. Hence, the association between JAK inhibitors and VZV reactivation is biologically plausible, as these therapies mimic natural viral evasion mechanisms.

Regarding individual agents, the greater JAK1 selectivity of upadacitinib has been hypothesized to confer a lower risk of HZ compared with less selective inhibitors, such as baricitinib (JAK1/JAK2 selective) and tofacitinib (pan-JAKi) [5,12,29,33]. In accordance with this hypothesis, IRs in our cohort were numerically lower with upadacitinib. However, CIs overlapped substantially, precluding any inference of a true safety advantage of upadacitinib. A more consistently described drug-related determinant of risk is dose, as higher JAKi doses are linked to increased HZ incidence [14,17,32]. In the present study, dose–response effects could not be assessed due to the limited number of events.

With respect to primary diagnosis, IRs were highest in patients with spondyloarthritis and the lowest among patients with inflammatory bowel disease. Although diagnoses were grouped to improve interpretability and mitigate limited data, CIs remained wide and differences were not statistically significant, precluding definitive comparisons between groups. This highlights the need for larger disease-spanning cohorts to enable more robust comparative analyses in real-world settings.

Patients who developed HZ were substantially more likely to have diabetes mellitus (34.8% vs. 11.2%) and CKD (26.1% vs. 3.7%), and both conditions remained independently associated with the outcome in multivariable analyses. These findings are biologically plausible and align with existing evidence linking both conditions to impaired immune response [34,35]. Diabetes is a well-established risk factor for HZ in the general population, likely reflecting impaired T-cell function associated with chronic hyperglycemia [36,37]. Chronic kidney disease has also been associated with increased susceptibility to HZ, particularly in advanced stages, which may reflect impaired adaptive and innate immunity driven by low-grade chronic inflammation and uremic toxins [34,38,39]. However, both comorbidities have been less consistently evaluated and recognized in JAKi-treated populations compared with more established demographic and treatment-related risk factors. Our findings suggest that these conditions may help identify patients at increased risk for HZ when considering JAK-inhibiting therapies, warranting further investigation to confirm this relationship.

Interestingly, HSV infections requiring systemic antiviral therapy under JAKi exposure were significantly more frequent among patients who developed HZ. Although an increased susceptibility to HSV during JAKi treatment has been described [5,19,40], its link with subsequent HZ remains poorly explored. Nonetheless, this finding should be interpreted cautiously, as it may be influenced by differences in healthcare utilization and documentation, and is best regarded as hypothesis-generating.

In contrast, despite being reported as key risk factors in the literature [17,41], age, sex and glucocorticoid exposure were not significantly associated with HZ in this cohort. This likely reflects limited statistical power to detect moderate associations due to the relatively small number of events, rather than evidence of an absence of effect. History of prior HZ showed a numerical trend towards increased incidence among patients who developed HZ in the present cohort, but significance was similarly unmet. This variable is likely to be underreported, given the lack of systematic documentation in clinical records, potentially attenuating a true association.

A key aspect of this study concerns the evaluation of preventive practices. Despite the availability of RZV, uptake was low. Vaccination was attempted in only 17.5% of patients, with fewer than 10% receiving at least one dose of RZV. According to the Centers for Disease Control and Prevention and Advisory Committee on Immunization Practices, a two-dose vaccination schedule with RZV is currently recommended in adults aged 19 years and older who are or will be immunosuppressed in the context of disease or therapy [42]. Thus, rather than lack of indication, the low uptake reflects both under-prescription and suboptimal adherence. Where documented, reasons for non-adherence were all related to financial constraints—an expected outcome, since RZV is not reimbursed in the study setting, underscoring the known importance of healthcare policy in vaccine implementation [26].

Importantly, attendance at a dedicated infectious risk consultation was significantly associated with higher vaccination uptake, particularly when the appointment was prior to initiation of JAK inhibitor therapy. In routine practice, preventive responsibilities are often fragmented, which may result in missed opportunities for vaccination [43]. Our observation suggests that structured infectious risk assessment when contemplating JAKi introduction may help bridge gaps between guideline recommendations and RZV implementation.

Notably, no HZ events occurred among patients who received at least one dose of RZV. While conclusions regarding vaccine effectiveness cannot be drawn given the limited number of patients and follow-up, this observation is reassuring and consistent with the established efficacy of RZV in immunocompromised populations [44], supporting proactive vaccination strategies. Future studies are warranted to better define optimal HZ prevention approaches, including the timing and long-term effectiveness of vaccination, as well as the potential role of antiviral prophylaxis in selected higher-risk populations.

All HZ episodes in this cohort resolved without long-term sequelae and permanent JAKi therapy discontinuation was uncommon, in line with previous reports [15,17,30,45]. Nevertheless, disease burden was not negligible, as approximately one quarter of the cases were classified as severe, including disseminated disease and ophthalmic involvement. All six severe events occurred in older patients with rheumatologic disease, predominantly with comorbidities and additional immunosuppressive exposure. Although the small number of events precludes firm assertions, these observations add to the hypothesis that such factors may influence not only susceptibility to HZ occurrence but also severity of HZ [46].

This study has several limitations. Its retrospective design relying on EHR introduces the possibility of incomplete documentation and underreporting of variables under study, which may vary across medical specialties. The relatively small number of HZ events, together with the limited sample size in certain subgroups, particularly spondyloarthritis, limits the statistical power for subgroup comparisons. These analyses should therefore be interpreted as exploratory. The study was not powered to formally assess the independent contribution of specific IMIDs to herpes zoster risk, precluding its inclusion in multivariable modeling. The single-center design may affect generalizability of the findings and the inclusion of a heterogeneous population with multiple IMIDs may have introduced clinical variability that is not fully captured. In addition, the dynamic nature of JAKi complicates the attribution of risk to individual agents. Finally, the low vaccination uptake and limited follow-up after RZV administration prevent robust assessment of vaccine effectiveness.

Despite these limitations, this study has noteworthy strengths. Including a broad, multi-IMID cohort and accounting for person-time exposure across different JAK inhibitor treatments, it provides a comprehensive real-world perspective on HZ risk. It also offers a detailed characterization of comorbidities, treatment patterns, clinical outcomes and preventive practices. This integrated perspective is essential, as the challenge of HZ in JAKi-treated patients lies both in identifying individuals at higher risk and implementing effective and timely preventive strategies.

## 5. Conclusions

This study confirms that HZ remains a clinically relevant concern of JAKi therapy in patients with IMIDs in real-world practice, with IR higher than that observed in the general population and IMID patients not exposed to JAKi. The identification of diabetes mellitus and CKD as significant predictors of HZ may help identify individuals at higher risk. Furthermore, structured infectious risk assessment was associated with improved uptake of RZV, which showed encouraging signs towards protection against HZ.

Taken together, these findings support a proactive, preventive-focused approach in patients initiating JAKi therapy, particularly in those with a higher comorbidity burden and in settings where vaccination uptake remains suboptimal. Larger real-world studies spanning multiple IMIDs are warranted to enable robust comparisons across individual diagnoses and JAKi, clarify risk factors and further evaluate HZ vaccination strategies in this population.

## Figures and Tables

**Table 1:** Incidence rate of herpes zoster according to Janus kinase inhibitor use.

JAK Inhibitor	PY	HZ	IR/100 PY (95% CI)
Upadacitinib	310.7	10	3.22 (1.22–5.21)
Baricitinib	117.2	6	5.12 (1.03–9.21)
Tofacitinib	137.6	7	5.09 (1.31–8.86)

Abbreviations: CI—Confidence interval; HZ—Herpes zoster; IR—Incidence rate; JAK—Janus kinase; PY—Person-years.

**Table 2:** Incidence rate of herpes zoster according to disease group.

Disease Group	PY	HZ	IR/100 PY (95% CI)
Rheumatoid arthritis	265.3	10	3.77 (1.43–6.11)
Spondyloarthritis	53.3	4	7.51 (0.15–14.86)
Inflammatory bowel disease	159.4	4	2.51 (0.05–4.97)
Dermatological	87.6	5	5.70 (0.71–10.70)

Abbreviations: CI—Confidence interval; HZ—Herpes zoster; IR—Incidence rate; PY—Person-years.

**Table 3:** Demographic, clinical and treatment characteristics according to herpes zoster occurrence.

Variable	No HZ (*n* = 269)	HZ (*n* = 23)	*p*-Value
Age at JAKi initiation, median (IQR)	44 (31–56)	49 (39–58)	0.240
Female sex, *n* (%)	171 (63.6)	17 (73.9)	0.320
Primary diagnosis Rheumatoid arthritis, *n* (%) Spondyloarthritis group, *n* (%) Inflammatory bowel disease, *n* (%) Dermatological diseases, *n* (%)	100 (37.2) 33 (12.3) 87 (32.3) 49 (18.2)	10 (43.5) 4 (17.4) 4 (17.4) 5 (21.7)	0.428
Diabetes mellitus, *n* (%)	30 (11.2)	8 (34.8)	0.005
Chronic kidney disease, *n* (%)	10 (3.7)	6 (26.1)	<0.001
Prior HZ, *n* (%)	11 (4.1)	3 (13.0)	0.088
HSV requiring systemic antivirals, *n* (%)	11 (4.1)	4 (17.4)	0.022
Initial JAK inhibitor Upadacitinib, *n* (%) Baricitinib, *n* (%) Tofacitinib, *n* (%)	152 (56.5) 63 (23.4) 54 (20.1)	8 (34.8) 7 (30.4) 8 (34.8)	0.110
Number of JAKis received 1, *n* (%) 2, *n* (%)	231 (85.9) 38 (14.1)	20 (87.0) 3 (13.0)	1.000
Days of JAKi exposure, median (IQR)	532 (333–919)	708 (306–978)	0.500
No. of concomitant immunomodulatory agents * 0, *n* (%) 1, *n* (%) 2+, *n* (%)	122 (41.8) 97 (36.1) 50 (18.6)	12 (52.2) 7 (30.4) 4 (17.4)	0.811
Corticosteroid use, *n* (%) Prednisolone ≥ 7.5 mg/day	124 (46.3) 48 (17.8)	10 (43.5) 5 (21.7)	0.797 0.582

Abbreviations: HSV—Herpes simplex virus; HZ—Herpes zoster; IQR—Interquartile range; JAKi—Janus kinase inhibitor. * This number accounts for systemic corticosteroids, conventional and biological disease-modifying agents.

**Table 4:** Multivariate Cox regression for risk of herpes zoster.

Variable	Hazard Ratio	95% CI	*p*-Value
Chronic kidney disease	3.24	1.17–8.95	0.023
Diabetes mellitus	3.05	1.28–7.29	0.012

Abbreviation: CI—confidence interval.

**Table 5:** Demographic and clinical characteristics according to uptake of at least one dose of recombinant zoster vaccine.

Variable	No RZV (*n* = 263)	RZV (*n* = 29)	*p*-Value
Female sex, *n* (%)	175 (66.5)	13 (44.8)	0.020
Age at JAKi initiation, median (IQR)	44 (31–55)	49 (32–59)	0.293
Primary diagnosis - Rheumatoid arthritis, *n* (%) - Spondyloarthritis group, *n* (%) - Inflammatory bowel disease, *n* (%) - Dermatological diseases, *n* (%)	105 (39.9) 34 (12.9) 76 (28.9) 48 (18.3)	5 (17.2) 3 (10.3) 15 (51.7) 6 (20.7)	0.043
Dedicated infectious risk consultation - IRC prior to JAKi initiation, *n* (%) - IRC post JAKi initiation, *n* (%)	73 (27.8) 51 (19.4) 22 (8.4)	16 (55.2) 14 (48.3) 2 (6.9)	0.002 <0.001 1.000

Abbreviations: IRC—Infectious risk consultation; IQR—Interquartile range; JAKi—Janus kinase inhibitor; RZV—Recombinant zoster vaccine.

**Table 6:** Characteristics of patients with complicated and recurrent herpes zoster.

Sex	Age	Disease	JAKi	CKD	DM	Previous HZ	DMARDs	PDNeq (mg/d)	Complication	Hospital Admission
M	64	RA	Upa 15 mg	No	Yes	No	-	0	HZO	No
F	70	RA	Upa 15 mg	Yes	Yes	Yes	-	5	Disseminated	No
F	65	RA	Bari 4 mg	Yes	No	No	-	5	Disseminated	No
F	58	RA	Bari 4 mg	No	No	No	MTX	7.5	Disseminated Bacterial SI	Yes
F	50	RA	Upa 15 mg	Yes	No	Yes	-	0	HZO	No
F	70	PsA	Tofa 5 mg bid	Yes	No	No	MTX	5	Disseminated	No
F *	40/43	PsA	Tofa 5 mg bid	No	No	No	-	0	Recurrence (×2)	No

Abbreviations: Bari—Baricitinib; bid—twice a day; CKD—Chronic kidney disease; DM—Diabetes mellitus; DMARDs—Disease-modifying antirheumatic drugs; F—Female; HZ—Herpes zoster; HZO—Herpes zoster ophthalmicus; JAKi—Janus kinase inhibitor; M—Male; MTX—Methotrexate; PDNeq—Prednisolone equivalent; PsA—Psoriatic arthritis; RA—Rheumatoid arthritis; SI—Superinfection; Tofa—Tofacitinib; Upa—Upadacitinib. * Data corresponds to two episodes of non-complicated herpes zoster after JAKi reintroduction.

## Data Availability

The data presented in this study are available on request from the corresponding author upon reasonable request.

## Supplementary Materials

The following supporting information can be downloaded at https://www.mdpi.com/article/10.3390/jcm15103733/s1. Supplementary Table S1: Distribution of patients and herpes zoster cases according to specific diagnosis; Supplementary Table S2: Distribution of concomitant immunomodulatory therapies in the overall cohort and in patients with herpes zoster; Supplementary Table S3: Janus kinase inhibitor regimens at treatment initiation and at herpes zoster diagnosis.

## Abbreviations

The following abbreviations are used in this manuscript:

CI	Confidence interval
CKD	Chronic kidney disease
EHR	Electronic health records
HR	Hazard ratio
HSV	Herpes simplex virus
HZ	Herpes zoster
IFN	Interferon
IMIDs	Immune-mediated inflammatory diseases
IQR	Interquartile range
IR	Incidence rate
IRC	Infectious risk consultation
JAKi	Janus kinase inhibitor
JAK-STAT	Janus kinase—signal transducer and activator of transcription
PY	Person-years
RZV	Recombinant zoster vaccine
VZV	Varicella-zoster virus
