# Supplementary material for: Herpes Zoster in Patients Treated with JAK Inhibitors for Immune-Mediated Inflammatory Diseases: Incidence, Associated Factors and Vaccination Uptake in a Real-World Cohort
Source: J Clin Med. 2026 May 13;15(10):3733. doi: 10.3390/jcm15103733 (PMC13207276; doi:10.3390/jcm15103733)
Supplement: Supplementary file 1 [file jcm-15-03733-s001.zip › jcm-4273627-supplementary.pdf]

Supplementary Materials

Supplementary Table S1. Distribution of patients and herpes zoster cases according to specific diagnosis.

Diagnosis	Patients (n=292)	HZ within diagnosis
Rheumatoid arthritis, n (%)	110 (37.7)	10 (9.1)
Ankylosing spondylitis, n (%)	20 (6.8)	2 (10.0)
Psoriatic arthritis, n (%)	17 (5.8)	2 (11.8)
Crohn disease, n (%)	37 (12.7)	1 (2.7)
Ulcerative colitis, n (%)	54 (18.5)	3 (5.6)
Atopic dermatitis, n (%)	38 (13.0)	2 (5.3)
Alopecia areata, n (%)	16 (5.5)	3 (18.8)

Abbreviations: HZ: Herpes zoster.

Supplementary Table S2. Distribution of concomitant immunomodulatory therapies in the overall cohort and in patients with herpes zoster.

Drug	Patients (n=292)	HZ within exposed patients
Methotrexate, n (%)	41 (14.0)	4 (9.8)
Leflunomide, n (%)	33 (11.3)	1 (3.0)
Azathioprine, n (%)	5 (1.7)	0 (0.0)
Cyclosporine, n (%)	3 (1.0)	0 (0.0)
Vedolizumab, n (%)	1 (0.3)	0 (0.0)

Abbreviations: HZ: Herpes zoster.

Supplementary Table S3. Janus kinase inhibitor regimens at treatment initiation and at herpes zoster diagnosis.

Drug	Initial therapy (n = 292)	At the time of HZ (n = 23)
**Upadacitinib, n (%)**		
15 mg	95 (32.5)	6 (26.1)
30 mg	8 (2.7)	3 (13.0)
45 mg	57 (19.5)	1 (4.3)
**Baricitinib, n (%)**		
2 mg	3 (1.0)	0 (0.0)
4 mg	67 (22.9)	6 (26.1)
**Tofacitinib, n (%)**		
5 mg, alternate days	0 (0.0)	1 (4.3)
5 mg id	19 (6.5)	0 (0.0)
5 mg bid	38 (13.0)	5 (21.7)
10 mg bid	5 (1.7)	1 (4.3)

Abbreviations: id – once a day; bid – twice a day; HZ: Herpes zoster.
